# Preparing for the Second Surge: Preventing Posttraumatic Stress Disorder and Building Resilience for Health Care Workers in the Face of COVID-19

**DOI:** 10.1017/dmp.2020.371

**Published:** 2020-10-13

**Authors:** Todd L. Benham, Alexander Hart, Michelangelo Bortolin, Michael Court, John Groves, Anthony Kraus, Brad Newbury, Amalia Voskanyan, Madeline Yogman, Fahad AlHajjaj, Yousef AlMalki, Bader Alossaimi, Oluwafunbi Awoniyi, Adham Sameer Bardeesi, Srihari Cattamanchi, Bridget Edwards, Anthony Hernandez, Fadi Issa, Philip Manners, Michael Molloy, Douglas Romney, Debra Weiner, Gregory R. Ciottone

**Affiliations:** Beth Israel Deaconess Medical Center Fellowship in Disaster Medicine COVID-19 Taskforce, Beth Israel Deaconess Medical Center, Brookline, MA, USA

**Keywords:** Coronavirus Disease 2019, COVID-19, healthcare workers, Posttraumatic Stress Disorder, resilience

## Abstract

The global community needs to be aware of the potential psychosocial consequences that may be experienced by health care workers who are actively managing patients with coronavirus disease (COVID-19). These health care workers are at increased risk for experiencing mood and trauma-related disorders, including posttraumatic stress disorder (PTSD). In this concept article, strategies are recommended for individual health care workers and hospital leadership to aid in mitigating the risk of PTSD, as well as to build resilience in light of a potential second surge of COVID-19.

Health care workers on the frontlines of the 2019 coronavirus disease (COVID-19) battlefield are facing a formidable enemy. While current efforts are focused on managing those patients who are being treated for COVID-19, the psychosocial burdens experienced by health care workers in the midst of the current pandemic and for the foreseeable future are an even more elusive enemy. The potential to devastate a generation of health care workers is substantial, particularly for those workers who provide care in facilities with a higher volume of patients with COVID-19 where exposure to distressing and traumatic situations is highly likely. Health care workers are at an increased risk for developing mental health conditions, and, as the intensity of the occupational stresses increases, impairments in social and occupational functioning are also likely to increase. These risks can be mitigated by health care workers and their leadership focusing on preventive wellness and self-care training, routine assessment of worker well-being, and incorporating strategies to assist health care workers in managing their reactions to traumatic events.

The suicides of a prominent New York City emergency physician and a nurse in Italy, both of whom have been on the frontlines of the COVID-19 response, are tragic indicators that the traumatic events incurred by many health care workers during this pandemic have overwhelmed their ability to overcome their distress.^1,2^ As expected, psychological conditions have been routinely observed in the aftermath of prior disasters and disease outbreaks. Posttraumatic stress disorder (PTSD) is 1 such condition that has been demonstrated to impact disaster survivors, including health care workers.

For the general population, the COVID-19 pandemic in and of itself would not meet the American Psychiatric Association’s definition of a traumatic event. Specifically, the Diagnostic and Statistical Manual of Mental Disorders, 5th Edition (DSM-5) defines a traumatic event as “actual or threatened death, serious injury, or sexual violence.”^3^ In this sense, identifying the COVID-19 pandemic as a “traumatic event” is incumbent on an individual’s experiences during the pandemic. Exposure to a traumatic event requires that the person either directly and personally experiences the trauma; witnesses the trauma in-person as it occurred to others; learns that the trauma occurred to a close family member or friend; or experiences “repeated or extreme exposure to aversive details of the traumatic event.” Of note, “extreme exposure” does not include exposure to the trauma through media sources unless the exposure is work-related (eg, first responders, medical personnel).^3^ Given this definition, health care workers in facilities with a high-volume of patients with COVID-19 have a greater risk of exposure to a traumatic event.

The World Mental Health Survey Consortium suggests that over 70% of adults worldwide will be exposed to a traumatic event that meets the DSM-5 definition.^4^ In the United States, upwards of 90% of people are estimated to have at least 1 exposure to a traumatic event during their lifetime.^5^ Despite this fact, most people will not develop PTSD. The National Comorbidity Survey Replication estimated the lifetime prevalence of PTSD among adults in the United States to be 6.8%.^6^ Research on the prevalence of PTSD in health care workers is quite variable; however, nearly every study indicates that rates are significantly higher when compared with those of the general population. A study of nurses at the University of Colorado Hospital found that 18% met diagnostic criteria for PTSD.^7^ In Spain, 20% of health care workers in both general pediatric facilities and pediatric intensive care units (ICUs) reported having PTSD.^8^ A meta-analysis of 9 studies reported a PTSD prevalence of 14.8% among physicians.^9^ Moreover, physicians commit suicide at higher rates than do people in the general population and they avoid seeking professional assistance due to stigma and to concerns that seeking assistance may impact their professional licensure.^10^

## IMPACT OF TRAUMA ON HEALTH CARE WORKERS

A survey of over 500 hospital workers in Beijing indicated that 10% of those surveyed reported high levels of posttraumatic stress 3 years after the severe acute respiratory syndrome (SARS) outbreak.^11^ A cross-sectional, survey-based study of 1257 health care workers in China demonstrated that women, nurses, and frontline health care workers are the classes of health care professionals most affected by psychological burdens.^12^ Moreover, a recent review indicates that the severity of psychological symptoms is significantly influenced by age, gender, occupation, specialization, type of activities performed, and proximity to COVID-19 patients.^13^ Initial studies of health care workers on the front line of the COVID-19 pandemic are demonstrating that, oftentimes, greater than 50% of these health care workers are experiencing symptoms of depression, anxiety, or PTSD.^14,15^ If similar outcomes continue to occur in health care workers on the front line of the COVID-19 pandemic, it is possible that thousands of health care workers around the world would experience posttraumatic stress symptoms consistent with PTSD in the upcoming months and years. This possibility is tragic in its own right, but if a second surge of COVID-19 hospitalizations and deaths occurs, the decreased functioning and potential loss of hundreds or thousands of health care workers will be detrimental to global health care.

Several studies have indicated that people who have gone through a traumatic event will experience symptoms of PTSD almost immediately after the event has occurred.^16,17^ Secondary to a traumatic event, it is common to have unwanted re-experiencing of the event (eg, flashbacks, dreams); persistent avoidance of that which relates to or reminds one of the trauma; negative changes in thought processes and/or mood; and significant changes in physical or emotional arousal/reactivity associated with the trauma. Simply, a person who meets criteria for PTSD will have continued reminders of that traumatic event, creating distressing and overwhelming cognitive and emotional reactions, and resulting in active avoidance of such triggers. Distorted thoughts such as “I should have been able to save that patient” are often prevalent and typically result in mood disturbances or symptoms of significant hyperarousal and reactivity, such as anxiety, irritability, and hypervigilance. Together, these symptoms can cause significant difficulties in the health care worker’s social and occupational functioning.

A health care worker who is having difficulty dealing with the impact of a traumatic event will likely experience reminders of the trauma based on any number of triggers, such as images, thoughts, and sensory experiences. Although some triggers resulting in distressing thoughts or emotions might be easily identifiable, it is also likely that health care workers will have difficulty recognizing how certain situations or behaviors are related to their previous traumatic events, and how their interpretation of the trigger is impacting their mood and behavior.

As an example, a health care worker who has experienced an emotionally charged death of a patient may engage in inappropriate self-blame or blame of others about the cause of the death. These unrealistic beliefs may result in the development of a lack of trust in herself or himself and others, a sense of helplessness, or a strong belief that the health care worker must control everything. These or similar beliefs are likely to result in the health care worker experiencing anxiety, anger, and depression. Ultimately, these cognitive and emotional difficulties will likely result in interpersonal conflicts at work and home, and potential decrements to the quality of medical care provided.

## OPPORTUNITIES TO REDUCE DISTRESS AND PROMOTE RESILIENCE

The National Center for PTSD has described a comprehensive list of components that are recommended as essential tools for health care workers providing care during the pandemic.^18^ However, these recommendations are only as useful as the degree to which they are implemented. Starting or maintaining personal resilience goals requires that the health care worker be aware of these tools and have the motivation to either begin or continue these goals. A health care worker who has already been overwhelmed by a traumatic event will need to allow the expression of those emotions that naturally occur as a result of difficult or tragic circumstances. Moreover, health care workers will benefit from actively working to identify and address how they have been interpreting the cause of a traumatic event; how the event has impacted their personal belief systems; and ultimately how those beliefs have resulted in emotional distress and destructive or unhelpful behaviors.

In anticipation of a likely increase in cases of PTSD in the medical workforce, hospital and clinic leadership need to focus on increasing health care worker well-being throughout the course of the pandemic. When working through the clinical and administrative challenges consistent with a pandemic, leaders need to invite and listen to potential solutions from the entire team and then provide staff with the ability to make independent decisions. Leaders also need to identify aspects of clinic management (eg, managing surge capacity and mass fatalities) that create a higher propensity for emotional distress. Likewise, ensuring a collaborative and standardized process regarding end-of-life decision-making processes that includes a plan for managing the process during mass fatalities is likely to reduce health care worker distress.^19^

As daily monitoring for the presence of COVID-19 in health care workers is important, so too is a focus on preventive wellness and on routine assessment of health care worker well-being and mental health, including monitoring for PTSD. In most health care facilities, health care worker participation in mental health assessments would require voluntary participation. Screening assessments, such as the following, have been found useful in identifying potential mental health concerns: Patient Health Questionnaire-9, The Primary Care PTSD Screen for DSM-5 or the Posttraumatic Stress Checklist-5, Generalized Anxiety Disorder-7, and the Maslach Burnout Inventory.^20-24^ These screening instruments could be administered by mental health personnel already employed by an organization or by an external agency. Ideally, an organization would provide health care workers with the opportunity to complete these screening assessments during resilience and self-care training for its staff. Mental health personnel should be available during these training events, which should occur on a regular basis (no less than annually), be conducted at various times to accommodate workers on different shifts, and be increased in frequency during periods of increased stress. Administrators who personally model the importance of resilience and self-care for themselves and for their staff will demonstrate to the entire health care team that they have permission to engage in self-care. Practically speaking, during the COVID-19 pandemic, leaders need to increase their presence in the clinical environment, paying particular attention to how the team is responding to the physical and mental demands associated with providing care. During a prolonged pandemic response, organizations need to support their staff by ensuring that they provide basic necessities, such as food and hydration, as well as a work schedule and environment that not only allows, but also requires staff to take breaks where they can physically and emotionally reset. Ensuring that the staff take routine breaks is particularly important when they are having to wear personal protective equipment (PPE) for extended periods or managing multiple patients in an ICU setting.

Leaders should encourage health care workers to both support and rely on their colleagues. Increased opportunities for peer consultation/supervision and peer support groups can encourage health care workers to maintain their resilience practices. This type of support would likely be used more readily if health care teams incorporate daily huddles at the beginning and end of their shifts. Establishing a weekly or bi-weekly case consultation group would allow staff workers to openly discuss challenging cases or cases with a poor outcome. However, special attention should be made to ensure that these case consultation groups do not become attributional.

It is highly recommended that organizations provide readily available and easily accessible professional assistance for the health care team. A recommended option would be to integrate a mental health provider or religious support personnel with a treatment team, which would likely increase the likelihood of other team members seeking out the support personnel for routine resilience building skills as well as for support during a traumatic event. Finally, if health care workers demonstrate a decrease in functioning over several weeks, formal mental health treatment is strongly recommended.

In addition to providing resilience building strategies, screening, and interventions for health care workers, particular attention needs to be placed on providing assistance to non-white health care workers. While health care workers on the front lines of the COVID-19 pandemic are estimated to have 3 times the risk of having COVID-19 as compared with the general community, blacks, Asians, and minority ethnic health care workers, in particular, are at a risk 5 times that of the general community when compared with the non-Hispanic whites in a community.^25^ Moreover, the inappropriate use of or inadequate access to PPE are factors that increase risk of susceptibility to COVID-19. Non-white health care workers were significantly affected by limited access to PPE when compared with that of other health care workers.^25^

These findings are similar to other previous studies that suggest that non-white health care workers may be more likely to care for patients in facilities that have fewer resources,^26^ and in lower-income communities, where social distancing is more difficult and in populations that are at higher risk for COVID-19 and other medical conditions that exacerbate the symptoms of COVID-19. These conditions can create additional psychosocial burdens for non-white health care workers. Beyond improving access to and education on the use of PPE, non-white health care workers ought to be provided with additional training on resilience-building techniques with a special emphasis given to providing them with resources and time to engage in self-care.

## CONCLUSION

Increased exposure to recurrent traumatic events subsequently increases a health care worker’s risk of PTSD and can impair his or her professional and personal functioning. Assisting health care workers in managing their responses to these traumatic events is of paramount importance in mitigating their risk of developing a trauma-related disorder. Moreover, great efforts need to be made in assisting health care workers in building resilience, particularly as the health care system prepares for a potential second surge of COVID-19 infections. Hospital and clinic leadership can play a major role in providing avenues for health care workers to process their traumatic events, and building and reinforcing their resilience for the future.
